# Applicability of Geomorphic Index for the Potential Slope Instability in the Three River Region, Eastern Tibetan Plateau

**DOI:** 10.3390/s21196505

**Published:** 2021-09-29

**Authors:** Feng Liu, Xin Yao, Lingjing Li

**Affiliations:** Institute of Geomechanics, Chinese Academy of Geological Sciences, Beijing 100081, China; Yaox@cags.ac.cn (X.Y.); Lilj@cags.ac.cn (L.L.)

**Keywords:** geomorphic indices, potential landslide zone, DEM, Jinsha River

## Abstract

Geomorphic indices (e.g., the normalized channel steepness index (Ksn) and the stream length-gradient index (SL)) highlight changes in fluvial shapes and gradients. However, the application of these indices was seldom used to identify potential landslide zones. In this study, we used the Ksn and SL indices to detect the significant variations in the stream power along river reaches, which are anomalies associated with landslides, in the Zengqu River watershed, the upper reaches of the Jinsha River. Most of the landslide anomalies originate along the trunk and surrounding tributaries below the knickpoint of the mainstream. This suggests an erosional wave is migrating upstream throughout the drainage area. The fluvial incision may generate over-steepened hillslopes, which could fail in the future. In addition, the divide asymmetry index (DAI) predicts the direction of the divide as the headwaters migrate toward lower relief, higher elevation surfaces. Landslides are expected to occur as the unstable divide migrates. The proposed methodology can benefit the detection and characterization of potential landslide zones. It should improve hazard and risk analysis and the identification of drainage network areas associated with landslides.

## 1. Introduction

Landslides are highly stochastic geomorphological processes that shape the Earth’s landscapes [1,2], causing economic and life losses due to widespread spatial distribution [3]. The landslide hazard mitigation cannot do without the identification of the conditioning factors [4]. The use of optical remote sensing imaging for landslide detection is mainly based on various markers associated with the surface morphological features of the affected area [5,6,7]. However, it only provides synoptic information about the post-failure deformation surface features and lacks information about the previous deformation state [8]. The InSAR technique complements the abovementioned limitation of the landslide detection based on optical imagery [9,10]. Regardless of the type of remote sensing method, understanding the lithology, tectonics, topography, and climate is a fundamental prerequisite for landslide detection. Indeed, the interactions between tectonics, erosion, and climate change are a basic tenet of the tectonic geomorphology [11]. The surface processes associated with landslides can be investigated based on the river incision and drainage basin morphology in highly incised and rugged terrain. However, the method of studying the tectonic geomorphology has seldom been used to identify regions where landslides may occur along the valley sides of stream channels and watershed divide. For example, the anomalous values of the stream length-gradient (SL) index were used to analyze the change in the gradient along a channel affected by landslides [12,13,14,15]. However, they did not assess the potential regions that may be prone to landslides. Here, we focused on the Zengqu River, which is a tributary of the upper part of the Jinsha River. The region where landslides may occur was predicted via the geomorphic index method. This study aimed to provide a means of diagnosing potential hillslope failure on the valley side and drainage divide. The results of this study provide a possible hillslope deformation target for early landslide identification.

## 2. Study Area

**Figure 1:**
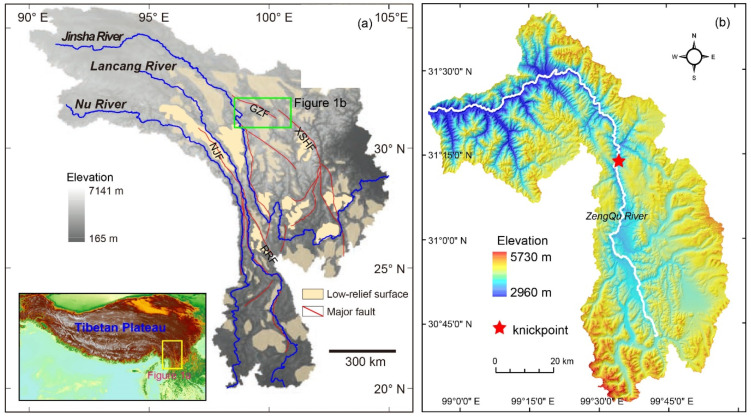
Topographic and fluvial characterization of the Three Rivers Region (TRR) (After Yang et al., 2015) (**a**) and the Zengqu River watershed (**b**). Abbreviations for some major faults: GZF: Ganzi fault; NJF: Nu Jiang fault; RRF: Red River fault; XSHF: Xianshuihe fault.

The Three Rivers Region (TRR) is located on the southeastern margin of the Tibetan Plateau (Figure 1a). The morphology of the TRR is characterized by the elongate, roughly parallel drainages of the Nu River, Lancang River, and Jinsha River. The tectonic activity in the TRR has mainly been characterized by strike-slip motion in the north and transtensional stress in the south during the Quaternary [16,17,18]. The most active, fault in the TRR is the Ganzi Fault system, a left-slip strike-slip system in the northern Jinsha watershed [19]. In addition, the active structures in the TRR also include a series of normal faults in the upper Jinsha River and Nu River watersheds [19]. The rocks are mainly sedimentary in the upper watersheds, and a mixture of sedimentary and deeply exhumed metamorphic rocks are exposed in the lower watersheds in the TRR [20]. The significant geomorphological feature is pervasive low relief, high-elevation topography, which is separated by incised canyons with high reliefs in SE Tibet [21,22,23]. In the TRR, the low relief, high-elevation surfaces are perched patches in the upper reaches (Figure 1a), while these surfaces have been completely eroded away in the lower watersheds [20].

**Figure 2:**
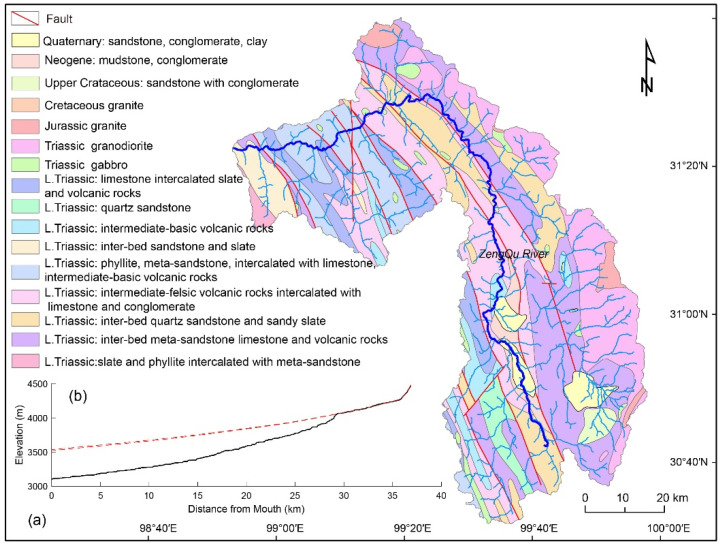
Simplified geological map (after 1:500,000 Chinese geological map) (**a**,**b**) reconstructed river long profile of the trunk stream of the Zengqu river extracted from the 30-m-resolution SRTM-DEM (derived from https://earthexplorer.usgs.gov/, accessed on 20 October 2020) (red dotted line is paleo-river profile and black line is modern river profile).

**Figure 3:**
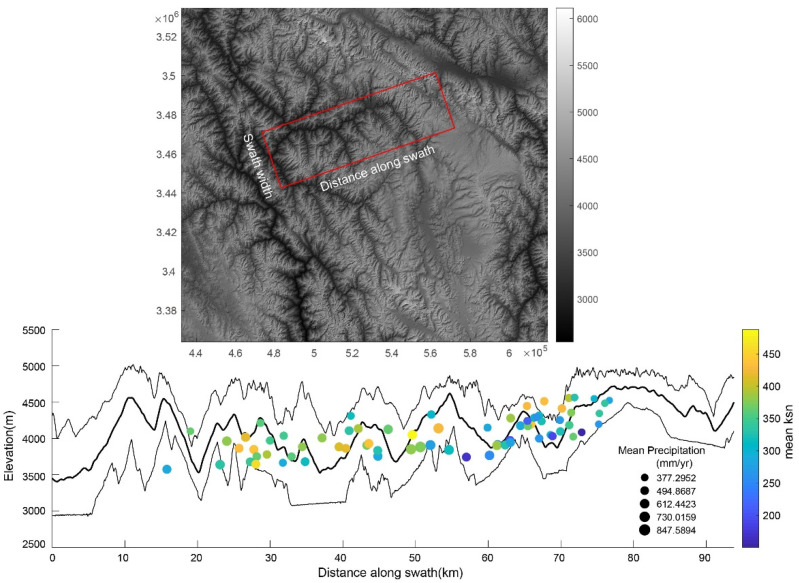
Swath profiles across the Zengqu River. (**Upper** plot): location of swath topographic profiles. (**Lower** plot): the black lines are maximum, mean, and minimum elevations along a 10-km wide swath from top to bottom, respectively. Mean annual averaged precipitation within basins (derived from TRMM data) in mm yr^−1^ colored by mean channel steepness.

The Zengqu River is a tributary of the upstream part of the Jinsha River (Figure 1b). It flows across different tectono-stratigraphic units and faults (Figure 2a). The lithological boundary and faults do not result in significant slope breaks in the longitudinal profile of the Zengqu River. The longitudinal profile indicates that the Zengqu River is in a transient state with a knickpoint (Figure 2b). Consequently, the channel is a deeply incised valley below the knickpoint. In contrast, the headwater of the Zengqu River has a lower relief. However, the precipitation does not exhibit a significant spatial gradient in the watershed due to the topographic effects (Figure 3).

## 3. Materials and Methods

**Figure 4:**
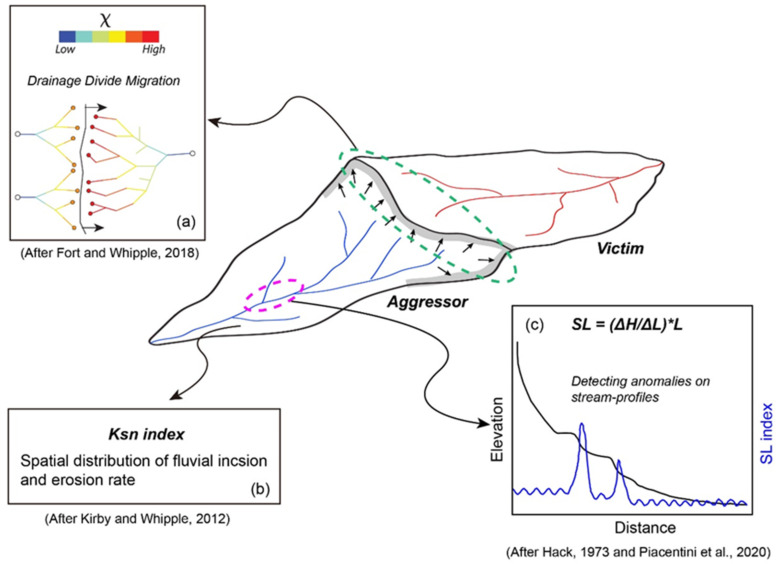
Cartoon plot show the methods adopted in this study on basis of basin scale (after [25]). (**a**) The parameter Χ provides a prediction of divide motion; (**b**) The Ksn index derived from Equation (1) indicate fluvial incision and associated with erosion rates; (**c**) The SL index (Equation (2)) highlights anomalous changes in river gradients.

The evolution of the topography is coupled to the changes in the river channel network in the TRR [24]. The normalized channel steepness index (Ksn), which is the slope of the channel normalized by the drainage area [25,26], was used to map the spatial changes in the fluvial incision capacity along the Zengqu River (Figure 4a). Because it is highly sensitive to changes in the channel gradient and can highlight the significant perturbations along a river [13], the SL index was used to detect the features of the surface processes associated with landslides in this study (Figure 4b). We assessed the relationship between the fluvial incision and the abnormal channel gradient based on the transient state of the Zengqu River. In the headwater region, the divide asymmetry index was used to determine whether the divide was stable and to predict the possible direction of the divide (Figure 4c). All indices can be calculated by the digital elevation model (DEM). We used the SRTM DEM (30 m resolution, derived from https://earthexplorer.usgs.gov/, accessed on 20 October 2020) to calculate the geomorphic indices. Based on these results, the regions where landslides may occur were identified.

### 3.1. Channel Steepness Index

The fluvial profiles and the channel steepness index are valuable tools for evaluating river incision in response to tectonics, climate, and rock properties [27,28,29,30]. Under steady-state or dynamic equilibrium conditions, under which uplift and erosion are spatially uniform, bedrock channels exhibit a power law relationship between the channel slope (*S*) and the drainage area (*A*) [31,32]:

*S* = *Ks*
*A*^−*θ*^ (1)

where *Ks* is the channel steepness index, and *θ* is the channel concavity index.

The above-mentioned power-law function is only valid for drainage areas above a critical threshold [33]. Generally, the minimum drainage area is determined by setting an area of 1 km^2^ when identifying the channel initiation points that separate the channels from the hillslopes [34]. The normalized steepness index (Ksn) allows the comparison of the steepness of channels in different drainage areas [29,34]. In addition, the Ksn values exhibit a meaningful correlation with the catchment-mean erosion rates determined from the concentrations of cosmogenic nuclides in river sediments [29,35,36]. Recently, the Caucasus case study revealed that the relative variations in the erosion rate could be inferred by combining the mean catchment normal channel steepness and the hillslope angle without the mean catchment erosion rate [37].

The uncertainties of the elevation data and the derived slope data may lead to unreliable channel steepness index results using the slope-area method [38]. Χ was proposed as a proxy map of the dynamic evolution of river networks with steady-state river geometry [38]. Under steady state conditions between uplift and erosion, the relationship between the channel elevation and Χ is expected to be linear, with a slope equal to the channel steepness [39]. Landslides can cause drainage divide migration [40], and erosion rates are balanced across divides in tectonically active landscapes [41,42]. However, the interpretation of χ-anomalies across a divide requires some caution due to the assumption of uniform uplift, rock erodibility, and climatic conditions, which may not occur in natural systems [39]. Previous studies have shown that χ-anomalies can occur even when a divide is immobile [27,43]. As an alternative metric of divide stability, Gilbert’s metrics, which includes the channel elevation, mean upstream gradient, mean upstream relief, and Χ, was proposed [41]. However, given the manual selection of divides and the across-divide comparisons in these studies, unwanted subjectivity was inevitably introduced [44]. Consequently, the divide asymmetry index (DAI) was proposed for the topographic analysis of divide migration [44]. The DAI is defined as the absolute value of the across-divide difference in the hillslope relief (ΔHR) normalized by the across-divide sum of the hillslope relief (ΣHR) [44]. The DAI ranges from 0 to 1, i.e., from entirely symmetric to mostly asymmetric, respectively. Thus, we used the DAI to identify the unstable divides in this study. Following the method described by Scherler and Schwanghart (2020), the DAI calculations in this study were conducted in TopoToolbox v2 [45].

### 3.2. SL Index

The SL index is another important geomorphic proxy that quantifies the stream profile’s steepness [44]. It is calculated using the following equation at the reach scale:

SL = (ΔH/ΔL)∗L,(2)

where L is the stream length measured from the headwaters to the reach midpoint, and ΔH/ΔL is the local stream gradient. The SL index is typically used to determine the gradient changes along the stream’s longitudinal profile associated with tectonic uplift, rock type, and/or surface processes [46,47,48]. However, the interpretation of the traditional SL index values is limited by the subjective choice of thresholds for defining the anomalous values along the channel profile [13,14]. Recently, hotspot and cluster analysis of the SL index (SL-HCA) was proposed to improve the visualization of the anomalous values in the identification of tectonic structures and large landslides [14]. The SLix toolbox can be used to obtain the SL index map via the SL-HCA method [48]. Indeed, the SL anomaly zones are associated with rapid changes in the channel gradient (e.g., knickpoint) along the longitudinal profile. For bedrock or mixed bedrock-alluvial channels, interactions between the changes in the bedrock lithology, tectonic structures, and mass movements and the streambed are the potential geological and geomorphological triggers of the anomalous SL values [14].

## 4. Results

**Figure 5:**
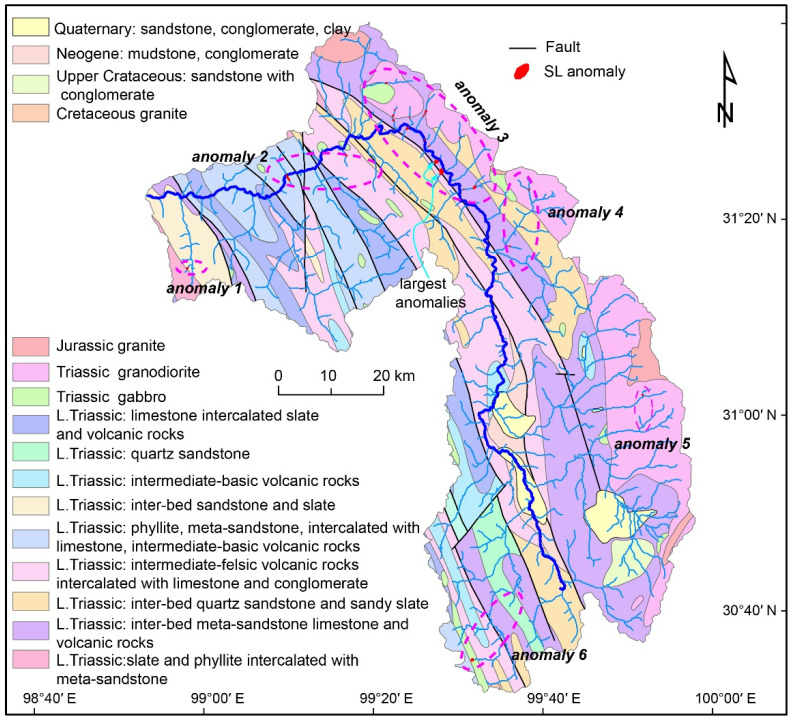
SL index anomalies and the catchment geology. The ellipses with magenta dashed line is significant SL index anomalous.

The SL-HCA anomaly map presents the distribution of the kernel density values of the SL index. Based on the threshold value equal to 1σ from the mean kernel density values of the SL index [14], the SL index anomaly map of the Zengqu River was produced (Figure 5). Based on this classification, we identified 30 SL-HCA anomalies in the study area. The anomalies were mainly concentrated in the tributary below the knickpoint in the Zengqu River and the two largest anomalies were located along the mainstream in anomaly region 3 (Figure 5). The anomalies were located at the boundaries between the lithological units in regions 1 and 6 (Figure 5).

**Figure 6:**
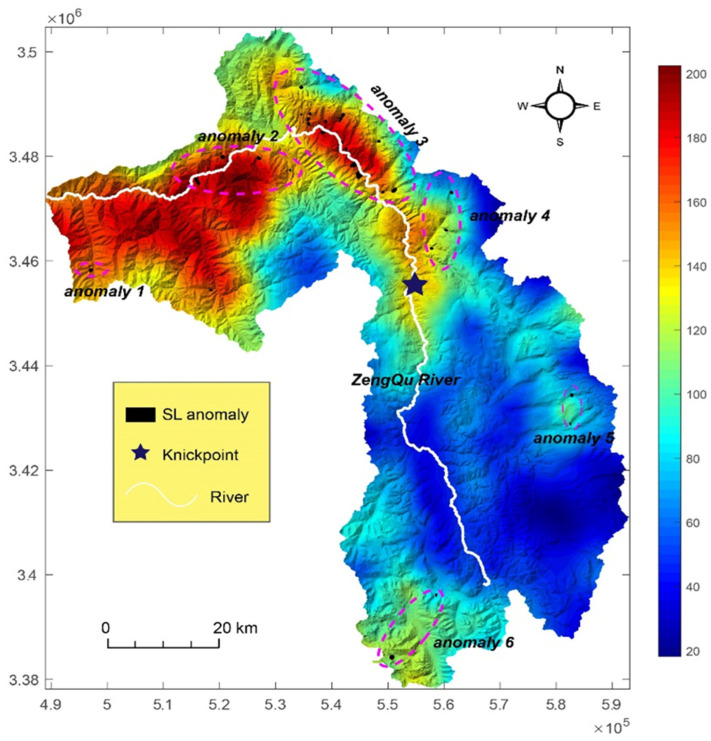
SL index anomalies and the spatial distribution of Ksn. The ellipses with magenta dashed line is significant SL index anomalous.

The regional distribution of the Ksn values was highly variable (Figure 6). The mean Ksn value varied from 20 to 200 m^0.9^. The high Ksn values were predominantly located in the northern part of the study area along the trunk of the Zengqu River. The strongest incision of the rivers occurs in the lower and middle reaches of the river, which is outlined by the spatial distribution of the Ksn.

**Figure 7:**
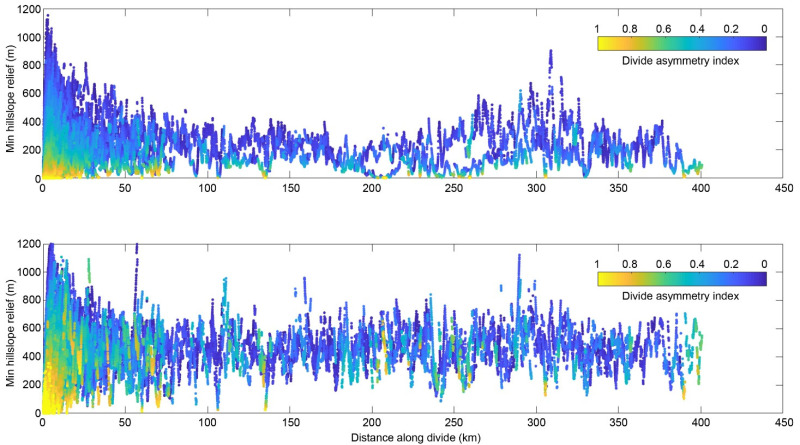
Minimum flow distance along the divide network of the Zengqu River catchment. Colors denote the divide asymmetry index (DAI).

**Figure 8:**
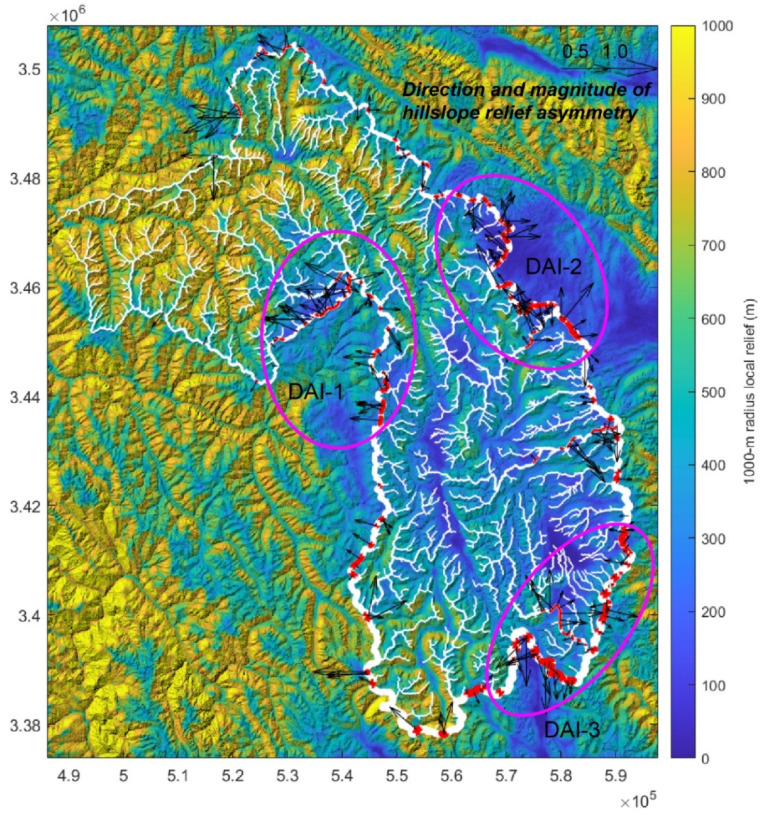
Anomalous divides in the Zengqu River catchment. White lines show the divide network, and red lines depict asymmetric (DAI > 0.4) divide edges. Black arrows indicate the direction and magnitude of the DAI, with the arrow pointing in the direction of the inferred direction of divide migration. The ellipses with the magenta line is significant anomalous divides.

Figure 6 shows how the DAI varied with distance along the divide network of the Zengqu River catchment. Some notable deviations occurred at divide distances of ~30–45, ~140, ~200–260, and ~380–400 km, and were typically associated with asymmetric divides (Figure 7). The most significant deviations from the average values occurred at divide distance of ~0–25 km. These potential migrated divides can be seen in the catchment boundary regions with significant local relief (Figure 8), and many clustered along the eastern and western edges of the catchment. The predicted migration direction is shown in Figure 8 based on the orientations of the divide segments and their mean DAI magnitude. Consequently, most of the divide migration would result in area loss of the Zengqu River catchment from higher to lower relief.

## 5. Discussion

### 5.1. SL-HCA Anomalies and Potential Landslide Zones

Generally, the anomalies detected using the SL-HCA method can be classified as three types related to landslides, lithology, and tectonics [12,47]. Based on the spatial distribution of the 30 anomalies identified in this study, we suggest that most of the anomalies are landslide-type anomalies. The anomalies related to faults are interpreted as having a tectonic origin [49]. The Zengqu River flows across several fault zones (Figure 2a). However, these faults do not result in obvious anomalies along the mainstream of the Zengqu River. This can be validated from the longitudinal profile of the Zengqu River (Figure 2b). In anomaly region 2, the two largest anomalies are distributed along the fault line, not along the mainstream of the Zengqu River (Figure 5). Tectonic anomalies are longer than those related to lithological contacts and landslides [49]. Thus, they probably have a tectonic origin. The anomalies located at the lithological boundaries are classified as having a lithological origin [13,14]. In anomaly regions 2 and 6, few of the anomalies are located at lithological boundaries (Figure 8). The changes in the bedrock’s resistance to fluvial erosion cause the morphology anomalies along the channel’s gradient. The landslide anomalies are frequently short in length [49]. The shorter the anomaly, the more likely it is to have a landslide origin [14]. Thus, we suggest that the remaining anomalies in the study area may be mainly associated with landslides.

The landslide anomalies may be related to the transient state of the Zengqu River. The longitudinal profile of the Zengqu River indicates that the main trunk is in a transient state (Figure 2b). This suggests that an erosional wave will migrate upstream throughout the drainage system [34,50] before the channel reaches a new steady state. During this process, the river incision will most likely predispose the hillslope to mass movement that could dam the valley and induce channel gradient anomalies. If this case is true, the region between anomaly 3 and the knickpoint is a potential landslide zone (Figure 5). Based on the spatial distribution, the channel exhibits significant incision from the outlet to anomaly region 2. However, few landslide anomalies were identified in this region. In contrast, a large quantity of landslide anomalies are distributed along the main trunk and the surrounding tributaries in the anomaly region 3 (Figure 5). This also suggests that anomaly region 3 has already transmitted the erosional wave to its tributaries. That is, the reach below anomaly region 3 is in a nearly steady state. The channel gradient anomalies are minimal or have already disappeared, which may not be captured by the SL-HCA method. As the knickpoint of the trunk migrates upstream over time, the base level of the channel and the surrounding tributaries will change in the zone above anomaly region 3. Consequently, the fluvial incision may generate over-steepened hillslopes that could fail in the future.

### 5.2. Divide Migration and the Potential Landslide Zones

The DAI can be used to predict the direction of the divide as the headwaters migrate toward lower relief, higher elevation surfaces (Figure 7). The across-divide difference in the relief is a more direct proxy for erosion, and divides are expected to migrate from high relief to low relief [43]. Figure 7 shows the three main unstable divide regions. We suggest that region DAI-2 is a potential landslide zone. The segment of the divide in region DAI-2 is adjacent to a large patch of low-relief surfaces (Figure 7). In the three rivers region, low-relief surfaces at high elevations are transiently produced as a result of the dynamic reorganization of the river networks [51]. They may survive for some time before being degraded or captured as streams erode inwards from their outer perimeters [52]. These low-relief surfaces suggest less exhumation due to lower long-term exhumation rates and/or shorter durations of fluvial incision [20]. The erosion rates derived from low-temperature thermochronometry exhibit variable gradients from downstream to upstream in the three rivers region [24]. A similar spatial pattern of erosion was found based on detrital 10Be data [20]. This regional context implies cross-divide contrasts in erosion, and thus an unstable divide may emerge in region DAI-2.

It should be noted that not all of the identified anomalous divides are in fact unstable and migrating with time according to the proxy of the hillslope relief, flow distance, and divide asymmetry [44]. For regions DAI-1 and DAI-3, we are not sure of the cross-divide contrasts in erosion due to the comparatively small area of the low-relief surface compared to that in region DAI-2. Thus, it is not easy to determine whether their divides are unstable. Our approach provides a means of identifying the potential failure of hillslopes due to river incisions on the watershed scale. However, it does not provide a quantitative map of when and where landslides may occur.

## 6. Conclusions

The SL index was used to investigate the abnormal gradient along the Zengqu River. We identified 30 anomalies distributed across the study area. Most of them are landslide anomalies. The landslide anomalies mainly cluster in the high fluvial incision area, below the main knickpoint of the Zengqu River. They occurred in response to the base level perturbation caused by the progression of erosion upstream. The fluvial incision may generate over-steepened hillslopes that could fail in the future as the knickpoint of the trunk migrates upstream over time. The DAI can be used to predict the direction of the divide as the headwaters migrate toward the lower relief, higher elevation surfaces. The across-divide difference in the relief is expected to cause landslides as the erosion migrates from high relief to low relief. The proposed methodology can benefit the detection and characterization of potential landslide zones.

## Data Availability

Not applicable.
